# HIV care continuum characteristics among people with opioid use disorder and HIV in Vietnam: baseline results from the BRAVO study

**DOI:** 10.1186/s12889-020-08538-3

**Published:** 2020-03-30

**Authors:** Caroline King, Le Minh Giang, Gavin Bart, Lynn Kunkel, P. Todd Korthuis

**Affiliations:** 1 Department of Biomedical Engineering, School of Medicine, Oregon Health and Science University, Portland, OR USA; 2 MD/PhD Program, School of Medicine, Oregon Health and Science University, Portland, OR USA; 3 Hanoi Medical University, Hanoi, Vietnam; 4 Hennepin Healthcare, Minneapolis, MN USA; 5 Portland State University-Oregon Health & Science University School of Public Health, Portland, OR USA; 6 Department of Medicine, Oregon Health and Science University, Portland, OR USA

**Keywords:** HIV care continuum, Opioid use, Vietnam, Anti-retroviral medication, HIV viral suppression

## Abstract

**Background:**

Little is known about patient characteristics that contribute to initiating antiretroviral therapy (ART) and achieving viral suppression among HIV people with opioid use disorder in Vietnam. The primary objective of this analysis was to evaluate associations between participant characteristics and the critical steps in the HIV care continuum of ART initiation and HIV viral suppression among people with opioid use disorder and HIV in Vietnam.

**Methods:**

We assessed baseline participant characteristics, ART status, and HIV viral suppression (HIV RNA PCR < 200 copies/mL) enrolled in a clinical trial of HIV clinic-based buprenorphine versus referral for methadone among people with opioid use disorder in Vietnam. We developed logistic regression models to identify characteristics associated with ART status and HIV viral suppression.

**Results:**

Among 283 study participants, 191 (67.5%) were prescribed ART at baseline, and 168 of those on ART (90%) were virally suppressed. Years since HIV diagnosis (aOR = 1.12, 95% CI 1.06, 1.19) and being married (aOR = 2.83, 95% CI 1.51, 5.34) were associated with an increased likelihood of current prescription for ART at baseline. Greater depression symptoms were negatively associated with receipt of ART (aOR = 0.97, 95% CI = (0.94, 0.9963)). In the HIV suppression model, once adjusting for all included covariates, only receipt of ART was associated with viral suppression (aOR = 25.9, 95% CI = (12.5, 53.8). In bivariate analyses, methamphetamine was negatively correlated with ART prescription (*p* = 0.07) and viral suppression (*p* = 0.08).

**Conclusion:**

While fewer than 90% of participants had received ART, 90% of those on ART had achieved HIV viral suppression at baseline, suggesting that interventions to improve uptake of ART in Vietnam are essential for achieving UNAIDS 90–90-90 goals in people who use heroin in Vietnam. Social determinants of health associated with ART and HIV viral suppression suggest that social support may be a key to facilitating both of these steps in the HIV care continuum.

## Background

Opioid use disorder (OUD) and human immunodeficiency virus (HIV) are intertwined epidemics worldwide; around one-third of all new HIV infections outside of sub-Saharan African occur among people who inject drugs [1, 2]. In Vietnam, people who use drugs experience even higher rates of HIV infection, with an estimated 60% of new HIV cases occurring among people who inject drugs [3]. In Vietnam, research is needed to explore what factors may impact aspects of the HIV care continuum, including anti-retroviral therapy (ART) initiation and viral suppression, among people with opioid use disorder and HIV.

In 2014, the Joint United Nations Program on HIV/AIDS released goals for HIV engagement and treatment by 2020: that 90% of all people living with HIV will know their status; 90% of all people diagnosed with HIV will receive antiretroviral therapy; and 90% of those on antiretroviral therapy will be virally suppressed [4]. Recent literature highlights systems-level factors that impact this care continuum in Vietnam, including the cost of treatment [5], care coordination, chronic disease management, and peer networks [6]. However, little is known about patient level factors that contribute to engaging in the HIV care continuum in Vietnam.

The Vietnamese government has worked to provide care through specialized yet separate clinics for HIV care and OUD over the past 15 years [7]. Beginning in 2005, Vietnam began providing antiretroviral therapy through the U.S. President’s Emergency Funding for AIDS Relief (PEPFAR) funding [7]. Today, of the approximately 250,000 people living with HIV in Vietnam, nearly 120,000 people receive ART; approximately half of those on ART now receive locally-funded care (versus external aid) [8]. Despite these gains, we extrapolate from these numbers that at least 130,000 people living with HIV remain unconnected or unengaged in care in Vietnam. Gaps remain in creating systems that identify, engage, and retain patients with HIV in care that meets their needs.

Vietnamese efforts to address injection drug use are multifaceted. For several decades, Vietnam has used compulsory drug detention centers (known as 06 centers) to curb substance use [9]. In 2008, Vietnam began to shift from this model, building clinics to provide methadone maintenance therapy (MMT) to people with opioid use disorder [10]. Following the success of initial MMT pilot demonstrations, the Vietnamese government expanded MMT clinics to all provinces in Vietnam, creating access to treatment for an estimated 50,000 people [10].

Over the past several years, changes in external aid challenged Vietnam to identify how to continue to provide HIV and opioid use disorder care in communities. Research completed in 2016 found most people with HIV and OUD in Vietnam prefer integrated treatment, where they can receive both MMT and ART at the same time [11]. Further data suggested that addressing both HIV and opioid use disorder treatment can enhance HIV outcomes and decrease behaviors that increase transmission risk [12–14]. Over the last several years, Vietnam has successfully piloted clinics that provide both HIV and MMT.

To date, few studies have explored what individual characteristics may contribute to viral suppression in Vietnam regardless of opioid use. Several studies demonstrate that comorbid substance use disorders can decrease the likelihood of HIV viral suppression [15, 16], though the majority of this effect is likely related to social barriers rather than a physiologic mechanism. One study found adherence to ART and use of trimethoprim/sulfamethoxazole as predictors of HIV viral suppression, while a history of tuberculosis was a risk factor for non-suppression [7]. In another study, younger age at beginning of treatment and positive Hep-C antibody were associated with virologic failure on HIV treatment in Vietnam [17]. Social isolation, high perceived stigma, and multiple daily pill regimens have also been associated with suboptimal HIV virologic suppression in Vietnam [18]. The primary objective of this analysis was to evaluate associations between participant characteristics and the critical steps in the HIV care continuum of ART initiation and HIV viral suppression (HIV-1 RNA PCR < 200 copies/mL) among people with opioid use disorder and HIV in Vietnam.

## Methods

### Study design and setting

This study reports cross-sectional baseline characteristics of the Buprenorphine to Improve HIV Care Engagement and Outcomes (BRAVO) Randomized Trial (ClinicalTrials.govNCT01936857). The BRAVO trial enrolled people with HIV and moderate-to-severe opiate use disorder to receive either methadone maintenance therapy (MMT) or buprenorphine/naloxone (BUP/NX) at six Vietnamese HIV clinics between 2015 and 2019. The study clinics were chosen in the epicenter of Vietnam’s HIV and opiate epidemics; four clinics are located in Hanoi, one in Tanh Hoa Province, and one in Bac Giang Province. Clinics were selected based on the high prevalence of untreated opiate use disorder among newly engaging people with a history of intravenous drug use, ability to enroll sufficiently for the study, availability of MMT, and support of local authorities.

This study was conducted in partnership with Hanoi Medical University, the Provincial AIDS Control authorities of Hanoi, Thanh Hoa and Bac Giang, the Vietnamese National Institute of Mental Health and Oregon Health & Science University in Portland, Oregon.

### Consent and data collection

Research assistants reviewed the written Vietnamese language informed consents with participants in a confidential space in the HIV clinic. Participants were instructed their study participation had no bearing on HIV treatment or other usual care at their clinic. Those agreeing to participate signed the consent form which was securely filed. Consents were periodically audited by the study quality assurance monitor for appropriate completion.

Research assistants administered surveys to participants in confidential settings using secured, web-based electronic data entry. All survey instruments were administered in Vietnamese and adapted for appropriateness during previous research. Blood specimens for HIV viral load PCR were collected at HIV clinics and sent to the National Hospital for Tropical Diseases in Hanoi, Vietnam for HIV-1 PCR testing using Abbott m2000rt RealTime HIV-1 PCR amplification assay [19].

### Participants

Participants were eligible for inclusion if they were HIV positive, had current moderate-to-severe DSM-V opioid use disorder (OUD), a positive urine drug screen for opioids at the time of enrollment, interest in receiving treatment for OUD, were age 18 or older, and were willing to practice birth control, if female. Study research staff prioritized patients new to HIV care or registered for care but not receiving ART, though participants already receiving ART were allowed to enroll.

Participants were ineligible if they had a known hypersensitivity to buprenorphine or naloxone, an aspartate aminotransferase or alanine aminotransferase greater than five times the upper limit of normal, were currently pregnant or breastfeeding, had a serious medical or psychiatric illness in the past 30 days that precluded safe participation in the opinion of the study physician, or received MMT within the past 30 days. Participants from known vulnerable populations, including decisionally-impaired adults, prisoners, and children, were also excluded.

### Measures

We assessed two critical steps in the HIV care continuum as study *outcome variables*: 1) ART initiation at time of enrollment, classified as a self-report of currently taking ART at the time of study enrollment, and 2) HIV viral suppression at time of enrollment, defined as HIV RNA PCR < 200 copies/mL.

Participant characteristics included gender (male/female), greater than 9th grade education (yes/no), currently employed (yes/no), currently married (yes/no), history of 06 rehabilitation (yes/no), substance use (positive urine drug screen (UDS) for methamphetamine or amphetamine, methadone, or buprenorphine), and self-reported daily tobacco use and risky alcohol use (defined as an AUDIT-C score greater than 3 in women or greater than 4 in men) [20–24]. Continuous covariates included self-reported age, lifetime number of arrests, years since HIV diagnosis, and the Depression Anxiety Stress Scales (DASS) [25] scores for depression, anxiety and stress symptoms, which has been validated for use in Vietnamese [26].

### Data analysis

#### Primary analysis

We assessed bivariate associations (t-tests, Pearson’s chi-squared tests, and Fisher exact tests) of participant characteristics and separately, ART initiation and HIV viral suppression. Potential covariates were selected based on a priori hypotheses and previous studies. We then conducted bivariate analyses among those covariates and the study outcome (viral suppression). We chose to include covariates in our model that had *p*-values of < 0.10 in bivariate analyses. Regardless of statistical significance, we retained age and years since HIV diagnosis each final model. Two separate multivariable logistic regression models estimated the relationship of baseline participant characteristics with study outcomes. We used an estimated 10 events per degree of freedom ratio to estimate the number of covariates we could include to avoid overfitting [27]. We evaluated our continuous covariates for linearity in the log-odds using Lowess scatter plots, and also evaluated covariates for multi-collinearity, using a cutoff of > 0.80 as a marker for covariate review. Finally, we used Hosmer-Lemeshow tests (*p* > 0.05) to evaluate model goodness of fit. We dropped participants missing both age and gender, and replaced the three participants missing viral load at baseline as non-suppressed.

#### Sensitivity analysis

After completing our primary analyses, we identified influential observations using Pregibon’s Delta-Beta statistic and removed observations with a value greater than 0.20 from the model. If the directionality of associations changed, we planned to report these results alongside primary study results.

## Results

**Fig. 1:**
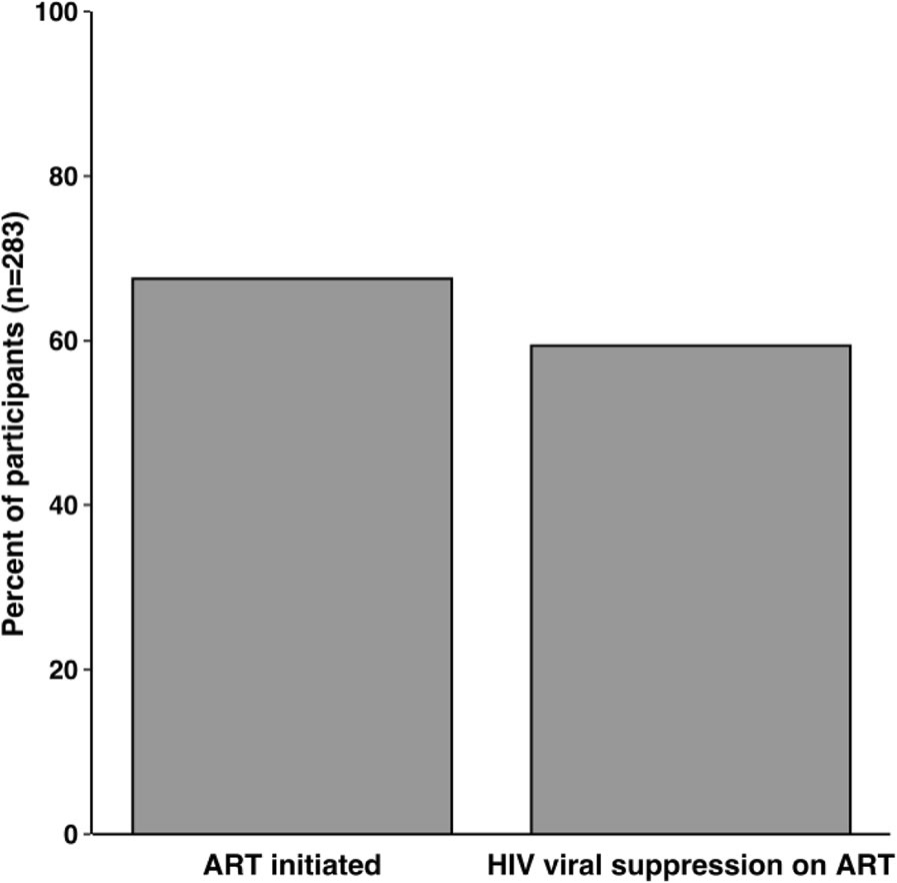
HIV care continuum among people with co-occurring opioid use disorder, 2015 to 2019

Participant baseline characteristics are reported in Table 1. Among the 283 study participants, most were men (96.8%) who smoke every day (82.0%), and have been arrested (82.7%) and sent to 06 rehabilitation at least once (60.8%). Of those included, 67.5% (191 of 283) were prescribed ART at baseline and 90% (168 of 191) of those on ART were virally suppressed. An additional 6.0% (17 of 283) of people were virally suppressed, but did not report taking ART, at baseline (Fig. 1).

**Table 1:** Unadjusted and adjusted associations between baseline characteristics and ART prescription and viral suppression among people with HIV and opioid use disorder in Vietnam, 2015–2019

	All participants (*n* = 283)	Prescribed ART *n* = 191/273 (70.0%)		Virally suppressed *n* = 189/283 (66.8%)	
	n (%)	Unadjusted n (%)	Adjusted aOR (95% CI)	Unadjusted n (%)	Adjusted aOR (95% CI)
**Mean Age (SD)**	38.3 (6.1)	*p = 0.102* 38.8 (6.0)	1.02 (0.97, 1.07)	*p = 0.02* 38.8 (6.0)	1.04 (0.98, 1.10)
**Gender (male)**	274 (96.8%)	*–*	–	–	–
**Greater than 9th grade education**		*p = 0.86*	–	*p = 0.84*	–
Yes	115 (40.6%)	77 (40.3%)		76 (40.2%)	
No	168 (59.4%)	114 (59.7%)		113 (60.0%)	
**Employed**		*p = 0.31*	–	*p = 0.12*	–
Yes	130 (45.9%)	92 (48.2%)		93 (49.2%)	
No	153 (54.1%)	99 (51.8%)		96 (50.8%)	
**Married**		*p = 0.003*	2.83 (1.51, 5.34)	*p = 0.004*	1.44 (0.69, 3.03)
Yes	109 (38.5%)	85 (44.5%)		84 (44.4%)	
No	174 (61.5%)	106 (55.5%)		105 (55.6%)	
**History of arrest** (*n* = 280)		–	–	–	–
Yes	234 (82.7%)				
No	46 (16.3%)				
**Lifetime number of arrests** (*n* = 282)	2.2 (1.7)	*p = 0.13* 2.3 (1.7)	–	*p = 0.17 2.3 (1.7)*	–
**History of 06 rehabilitation**		*p = 0.03*	1.58 (0.88, 2.85)	*p = 0.07*	1.14 (0.55, 2.34)
Yes	172 (60.8%)	125 (65.4%)		112 (59.3%)	
No	111 (39.2%)	66 (34.6%)		67 (35.4%)	
**Currently on ART** (*n* = 273)		–	–	*p < 0.001*	25.4 (12.13,
Yes	191 (67.5%)			168 (88.9%)	53.11)
No	92 (32.5%)			17 (9.0%)	
**Mean Years since HIV diagnosis (SD)**	7.4 (5.7)	*p = < 0.0001* 8.4 (5.4)	1.12 (1.06, 1.19)	*p = 0.002* *8.1 (5.5)*	0.99 (0.93, 1.06)
**Mean CD4 cell count (SD)**	405.0 (224.1)	*–*	–	*–*	–
**Daily tobacco use**		*p = 0.24*	–	*p = 0.52*	–
Yes	232 (82.0%)	154 (80.6%)		153 (81.0%)	
No	51 (18.0%)	37 (19.4%)		36 (19.0%)	
**Risky alcohol use** (*n* = 266)		*p = 0.14*	–	*p = 0.41*	–
Yes	90 (31.8%)	56 (29.3%)		53 (28.0%)	
No	193 (68.2%)	126 (66.0%)		130 (68.8%)	
**Positive UDS for Methamphetamines**^**a**^		*p =* 0.07	0.55 (0.28, 1.09)	*p* = 0.08	0.74 (0.32, 1.70)
Yes	53 (18.7%)	31 (16.2%)		30 (15.9%)	
No	230 (81.3%)	160 (83.8%)		159 (84.1%)	
**Positive UDS for methadone**		*–*	–	*–*	–
Yes	4 (1.4%)				
No	279 (98.6%)				
**Positive UDS for buprenorphine**		*–*	–	*–*	–
Yes	1 (0.4%)				
No	282 (99.6%)				
**DASS Anxiety Sub-score (SD)**	4.5 (8.3)	*p = 0.77* 4.42 (9.8)	–	*p = 0.99* 4.48 (9.8)	–
**DASS Stress Sub-score (SD)**	5.4 (12.1)	*p = 0.69* 5.7 (14.4)	–	*p = 0.57* 5.7 (14.5)	–
**DASS Depression Sub-score (SD)**	6.1 (10.2)	*p = 0.02* 5.1 (7.3)	0.97 (0.94, 0.9963)	*p = 0.07* 5.3 (7.4)	0.99 (0.96, 1.03)

^a^Includes methamphetamines and amphetamines

In bivariate analyses, increased age, number of years since HIV diagnosis, being married, and a history of 06 rehabilitation were associated with being on ART at study entry., while positive urine drug screen for methamphetamine/amphetamines and increased DASS depression sub-scores were associated lack of ART at study entry. Similarly, age, being married, history of 06 rehabilitation, and being on ART were associated with HIV viral suppression at study entry, while a positive methamphetamine/amphetamine urine drug screen, number of years since HIV diagnosis, and increased DASS depression sub-score were associated with a lack of HIV viral suppression at baseline (Table 1).

After adjusting for covariates, both years since HIV diagnosis (aOR = 1.12, 95% CI 1.06, 1.19) and being married (aOR = 2.83, 95% CI 1.51, 5.34) were associated with increased odds of being on ART at baseline. A higher DASS depression score was negatively associated with being on ART (aOR = 0.97, 95% CI 0.94, 0.9963). In the HIV viral suppression model, only current ART use remained associated with viral suppression (aOR = 25.9, 95% CI 12.5, 53.8) after adjusting for covariates. None of the sensitivity analyses identified changes in magnitude or direction of associations.

## Discussion

Our study indicates that the majority (67.5%) of people with OUD and HIV enrolling in the BRAVO study had already initiated ART at baseline; of those, 90% were virally suppressed, suggesting that further progress toward achieving the UNAIDS goal of 90% with HIV viral suppression in Vietnam hinges on increasing the proportion of people receiving ART. Inclusion of ART receipt in models of HIV viral suppression eclipsed the potential effect of other participants characteristics, such as methamphetamine use, supporting the WHO recommendation for offering ART to all persons living with HIV, regardless of drug use.

Vietnam is working to identify ways to help support people with HIV and co-occurring opioid use disorder in ways that work for these patients [11]. Our study suggests that once people are engaged in ART treatment, achieving viral suppression is highly feasible. Interventions to improve ART treatment initiation, such as integrating OUD treatment in HIV treatment settings, are urgently needed, particularly among those with shorter times since HIV diagnosis.

Being married and time since HIV diagnosis were associated with already being prescribed ART at baseline; increased depression symptoms were negatively associated with being on ART. The identification of marriage as associated with increased ART treatment initiation warrants further exploration into social supports and their role in aiding HIV treatment. Marriage may be a marker for greater social support in Vietnam, where the social fabric is tightly woven; family life is central to social connectedness in Vietnam. Married participants may also be responding to growing public health education on the importance of taking ART to reduce the chances of HIV transmission to uninfected partners [28]. To our knowledge, similar results have not been reported in Vietnam; however, research in China [29] and South Africa [30] has shown that marriage may be positively associated with ART initiation.

While depression is associated with suboptimal ART adherence [31] [32], studies are mixed on its potential association with ART initiation, with several studies demonstrating an absence of association [33–37]. This association has not, to our knowledge, been explored in Vietnam to date. Authors working in China suggest that subpopulations of patients with depression may have differing levels of ART initiation [36]; our study suggests that patients with increased depression symptoms were less likely to have initiated ART at baseline. This warrants further investigation, particularly as a recent study in Vietnam showed that one-fifth of patients living with HIV who were sampled endorsed depression symptoms [38]. Improved mental health care access and engagement may support goals for HIV treatment engagement in Vietnam.

Among study participants, the strong association between ART receipt and HIV viral suppression eclipsed other important participant characteristics associated with HIV viral suppression in bivariate analysis, yet suggests that increasing access to ART from 70% to the UNAIDS target of greater than 90% is likely to achieve HIV viral suppression in people who receives ART. This study finding should not be interpreted as supporting public health approaches that only support ART roll-out without attention to substance use disorder treatment. Rather, it is essential to support ongoing opioid use disorder prevention and treatment interventions in order to reduce medical comorbidity and non-AIDS related deaths among people with opioid use disorder who account for the majority of HIV infections in Vietnam [3]. Further research regarding the potential role of strong social supports and ART self-management practices in Vietnam may suggest ways to improve HIV viral suppression among people who inject drugs in other countries.

In bivariate analyses, age, marital status, and history of 06 rehabilitation were associated with viral suppression; a positive methamphetamine/amphetamine urine drug screen, years since HIV diagnosis, and increased DASS depression sub-score were associated with decreased odds of HIV viral suppression. Similarly to ART status, marriage here may reflect the strong social connections that are a pillar of Vietnamese culture. Studies in Italy [39] and Russia [40] suggest that marriage was associated with viral suppression in these communities; this may be because of the social support from these relationships.

Studies primarily conducted in the United States have demonstrated the negative association between co-occurring methamphetamine use and viral suppression [41–44]. Our finding in bivariate analyses maps onto this body of literature, and warrants further investigation in this potentially hard-to-reach population. Finally, higher rates of depression have also been associated with decreased viral suppression [45, 46]. Improved mental health care access and engagement may be critical in meeting UNAIDS 90–90-90 goals.

Our study findings should be interpreted in light of several potential limitations. First, we cannot make causal inferences between participant characteristics associated with being on ART at baseline due to the study’s cross-sectional design. Additional research is needed to evaluate participants characteristics associated with the HIV care continuum among this patient population over time. Second, we were not able to consider the many structural characteristics potentially associated with being on ART or HIV viral suppression. The BRAVO intervention, however, is a structural intervention to increase access to OUD treatment in Vietnam and will assessed prospectively. Third, Vietnam is a middle-income country that has devoted substantial national resources to increasing treatment for both HIV and OUD. Results may not be generalizable to countries without such interventions, but may serve as a guide for those wishing to expand care services.

## Conclusions

This study provides a snapshot of Vietnam’s recent strides to meet UNAIDS goals for HIV treatment initiation and viral suppression by 2020. Future research should identify additional patient-level factors associated with aspects of the HIV care continuum among people with opioid use disorder, including longitudinal interventions of integrated care models for treatment of HIV and OUD.

## Data Availability

The datasets used and/or analysed during the current study are available from the corresponding author on reasonable request and consent of the OHSU Institutional Review Board and the Vietnam Ministry of Health Research Ethics Committee.

## Abbreviations

ART: Anti-Retroviral Therapy
OUD: Opioid Use Disorder
UNAIDS: The Joint United Nations Programme on HIV/AIDS
HIV/AIDS: Human Immunodeficiency Virus/Acquired Immune Deficiency Syndrome
DASS: Depression Anxiety Stress Score
BRAVO: Buprenorphine to Improve HIV Care Engagement and Outcomes
UDS: Urine Drug Screen
PCR: Polymerase Chain Reaction
